# One-Year Outcomes for Depression and Anxiety in SLE Patients

**DOI:** 10.3390/biomedicines12030484

**Published:** 2024-02-21

**Authors:** Liliana Duca, Nadinne Alexandra Roman, Petru Ifteni, Andreea Teodorescu

**Affiliations:** 1 Faculty of Medicine, Transilvania University of Braşov, 500036 Braşov, Romania; liliduca@yahoo.com (L.D.); petru_ifteni@yahoo.com (P.I.); andreea.teodorescu@unitbv.ro (A.T.); 2 Department of Clinical Immunology, County Emergency Clinic Hospital, 500326 Braşov, Romania; 3 Clinical Hospital of Psychiatry and Neurology of Brasov, 500123 Braşov, Romania

**Keywords:** NPSE, anxiety, depression, medication, evolution, assessment

## Abstract

Neuropsychiatric Systemic Lupus Erythematosus (NPSLE) is a severe form of SLE involving the nervous system, resulting in neurological and psychiatric symptoms. Although research has shown that SLE patients often suffer from cognitive impairments, depression, and anxiety, there are no specialized guidelines for psychiatric assessment and treatment. This study aimed to investigate the progression of neuropsychiatric symptoms in SLE patients, explicitly focusing on anxiety and depression, over a year. It also aimed to identify potential biomarkers linked to NPSLE and explore the connection between NPSLE and the overall progression of SLE. Our research involved a longitudinal study with 65 adults diagnosed with SLE. Participants underwent various physical, biochemical, and serological tests and were assessed using disease activity indexes like BILAG-2004 and SLEDAI-2K. Participants also underwent psychological assessments using the Hamilton Anxiety and Depression Rating Scales. The study did not find any significant impact of antidepressant therapy on the evolution of anxiety and depression among participants. However, medications like Methotrexate and Plaquenil showed a substantial reduction in these symptoms. Moreover, anxiolytic therapy seems to influence depression in SLE patients. The study also noted that anxiety levels tend to increase over time but are not directly associated with SLE activity. This study concludes that although specific SLE medications can affect the level of anxiety and depression, the overall effectiveness of neuropsychiatric therapy in managing these symptoms is limited. The findings suggest that further research into the tailored management of NPSLE symptoms and a deeper understanding of the disease’s psychiatric aspects are needed.

## 1. Introduction

Systemic lupus erythematosus (SLE) is a disease that affects the brain and nervous system by causing immune system attacks, resulting in various neurological disorders [1]. One of the critical forms of this disease is Neuropsychiatric Systemic Lupus Erythematosus (NPSLE), which affects the nervous system and causes both neurological and psychiatric symptoms, often leading to a grim outlook and high fatality rates [2]. Previous research highlights the widespread occurrence of psychiatric symptoms in most individuals afflicted with SLE. However, a notable lack of specialized guidelines for their psychiatric assessment and treatment exists. The most common symptom is cognitive impairment, affecting up to 80% of patients with SLE, followed by depression, which is seen in about 39% of cases. Additionally, some studies suggest a higher incidence of anxiety disorders among these patients [3,4].

A study conducted in 2019 involving 69 studies showed that there is significant variation in the occurrence of depression and anxiety among individuals with SLE. The rates ranged from 8.7% to 78.6% for depression and 1.1% to 71.4% for anxiety. This wide range is likely due to inconsistencies in how depression and anxiety are measured and defined in the context of SLE [5]. In addition, mood disorders related to SLE are not only highly prevalent but also profoundly affect the patient’s well-being. They are associated with a higher risk of cardiovascular diseases such as heart attacks, disability, diminished quality of life, a heightened suicide risk, and early mortality [6,7,8,9,10,11,12].

The European League Against Rheumatism emphasizes the significance of meticulous evaluation for neurological symptoms in SLE patients, stressing the exclusion of other potential causes, including medication-induced symptoms [13]. Given these guidelines, identifying specific biomarkers for psychiatric symptoms in lupus is a priority, considering that the current diagnostic methods need refinement and staging [3]. NPSLE concerns complex pathophysiological mechanisms, including immune responses leading to vascular or neuronal damage, inflammatory cytokines accumulating in the cerebrospinal fluid, compromised blood–brain barrier integrity, and hastened atherosclerosis [14]. Research indicates a notable link between SLE-related psychiatric symptoms such as depression and anxiety and the presence of specific markers like anti-cardiolipin (ACL), anti-beta2 glycoprotein I antibodies (Anti B2 GP1), lupus anticoagulant (LA), intercellular adhesion molecule (I CAM-1), diminished C4, and anti-ribosomal P antibodies (Anti RIB P). This connection underscores the potential role of both autoimmune/inflammatory and ischemic/thrombotic processes in the manifestation of depression and anxiety in NPSLE [15].

The treatment strategies aimed at addressing mood disorders related to SLE mainly target symptoms and involve the use of medications like antidepressants, antipsychotics, and anti-anxiety drugs for psychiatric conditions. Treatments often include immunosuppressive agents such as azathioprine, cyclophosphamide, corticosteroids, and mycophenolate mofetil to reduce systemic inflammation. In cases of severe SLE, high doses of glucocorticoids and intravenous cyclophosphamide are typically the first-line treatments. Alternatives like plasma exchange, rituximab, or intravenous immunoglobulin are considered for resistant forms. Additionally, azathioprine and mycophenolate mofetil are frequently used to manage SLE and address mild to moderate neuropsychiatric symptoms [16,17].

Concerning the progression of anxiety and depression over time in relation to the effectiveness of symptomatic treatments and the progression of SLE in terms of activity and damage, data are limited, with most studies focusing on cross-sectional data. A recent longitudinal study from 2022, spanning four years, indicated that anxiety levels remained consistent over time, with racial disparities in the severity of anxiety being noted. This study also highlighted that SLE activity was not longitudinally linked to anxiety when managing depression and other factors [18]. Another study on the longitudinal progression of depression, covering a similar four-year period, found that a significant portion (61.2%) of patients exhibited consistent symptoms of depression or major depressive disorder as per the Center for Epidemiologic Studies Depression Scale—Revised (CESD-R) scores [19]. Since anxiety and depression seem to be the most encountered psychiatric manifestations of NPSLE [20], the study presented in this paper aimed to investigate the progression of anxiety and depression in patients with NPSLE over a year. Due to the limited data available on this topic and the various factors that can influence the progression of neuropsychiatric symptoms, we examined the potential biomarkers linked to NPSLE that may indicate thrombosis and inflammation. Furthermore, in this paper, we will explore the relationship between NPSLE and the overall progression of SLE, including disease activity, new manifestations of SLE, and any further damage occurring over a year. The study will also analyse responses to standard therapies.

## 2. Materials and Methods

A study involving 65 adults diagnosed with SLE based on either SLICC or ACR criteria was conducted. They had been suffering from the condition for at least six months before their participation. These participants were recruited from June 2019 to January 2020, and the study was conducted at the Department of Clinical Immunology at the Brasov County Emergency Clinical Hospital in Romania. Before the beginning of the study, each participant provided informed consent, duly authorized by the local ethics committee, ensuring compliance with all regional protocols.

At the beginning of the study, none of the participants had been diagnosed with NPSLE. To ensure the accuracy of the results, subjects with a history of alcohol substance abuse, personality disorder, or major psychiatric conditions and subjects with a cortisol medication dose > 20 mg/daily were excluded. No pulse therapy with intravenous methylprednisolone was performed during the observation period. Severe manifestations of NPSLE (seizures, cerebrovascular diseases, cognitive dysfunction, aseptic meningitis) were also an exclusion criterion for including patients in the study. During the observation period, no such severe manifestations occurred. The characteristics of the patients are presented in Table 1.

During the study, participants underwent clinical examination and laboratory assessments. These included detailed physical and biochemical tests, as well as serological tests specific to SLE. The tests included high-sensitivity C-reactive protein (hs-CRP), anti-Smith antibodies (Anti SM), anti-nuclear antibodies (ANA), anti-double-stranded DNA antibodies, beta(2)-glycoprotein 1 antibodies (Anti B2), anticardiolipin antibodies (ACL), lupus anticoagulant (LA), homocysteine, complement components C3 and C4, P-selectin, intercellular adhesion molecule (ICAM 1), plasminogen activator inhibitor type 1 (PAI 1), and anti-ribosomal P protein antibodies (Anti RIB P). For these serological tests, the ELISA Kit from Elabscience (Elabscience Bionovation Inc., Houston, TX, USA) was used. Normal ranges were set for each marker to ensure the accuracy of the results.

At the beginning of the study, the participants were evaluated to assess the activity of their SLE disease. After 12 months, the evaluation was conducted again using two indices: the British Isles Disease Activity Group 2004 Index (BILAG Index) and the Systemic Lupus Erythematosus Disease Activity Index (SLEDAI-2K). The SELENA-SLEDAI Flare Index (SFI) was used to identify SLE flares. Only participants without active disease were included in the study.

Based on the last four weeks’ disease activity, the BILAG-2004 index categorizes disease activity into five levels (A to E). It consists of 97 items, more than the original BILAG index, which had 86 items. After one year, participants’ scores were interpreted as follows: A (very active disease), B (moderate disease activity), C (mild and stable disease), D (no current activity but historical presence), and E (no current or past activity) [21]. The SLEDAI-2K is a sensitive tool that evaluates disease activity over the last 30 days and predicts mortality in SLE. It consists of 24 descriptors and scores more than what is considered for clinical significance [22].

The SELENA-SLEDAI Flare Index (SFI) was developed during the Safety of Estrogen in Lupus Erythematosus National Assessment (SELENA) trials. It integrates the SELENA-SLEDAI Flare Index, various levels of flares, and the Physician Global Assessment (PGA) of disease activity [23]. A SLEDAI score of 3 or more and at least a 1-point increase in the PGA (0–3 range) indicate a mild-to-moderate flare. A score of 12 or more on the SLEDAI, combined with an increase of 2.5 or more in the PGA, signifies a severe flare [24]. Additionally, we evaluated irreversible organ lesions with the Systemic Lupus International Collaborating Clinics (SLICC/ACR) damage index. The index was designed to assess permanent damage in patients with SLE, regardless of origin, with a maximum score of 47 [25]. Over time, the SLICC damage score tends to increase gradually, and studies have indicated that individuals with elevated impairment scores in disease progression are associated with a poorer prognosis and increased mortality [26].

We used the WHO Disability Assessment Schedule 2.0 to assess the levels of disability. Furthermore, we used the Hamilton Anxiety Scale (HAM A) and the Hamilton Depression Rating Scale (HAM D) to measure the anxiety and depression levels among the participants. Each patient showing changes in their depression and anxiety scores underwent an assessment by a psychiatric specialist. The specialist provided tailored treatment recommendations based on local guidelines. Therefore, 34 (52.31%) subjects did not receive any treatment, 4 (6.15) subjects received only anxiolytic treatment, 7 (10.77) patients were administered antidepressant therapy, and 20 (30.77) subjects received both anti-depressive and anti-anxiety medication.

Our statistical analysis was performed using IBM Statistical Package SPSS version 20.0 (IBM Corp. Released 2011. IBM SPSS Statistics for Windows, Version 20.0, Armonk, NY, USA). Linear regression was used to identify potential predictors for the neuropsychiatric manifestations (depression and anxiety) after a year of follow-up, consisting of disease manifestation, specific biomarkers for thrombotic and inflammatory manifestations, and psychiatric treatment. A univariate analysis of covariance (ANCOVA) was used to identify the influences of subjects’ characteristics, medication, and specific biomarkers on disease activity, anxiety, and depression at one-year follow-up. Values less than or equal to 0.05 were considered as significance cut-off points.

## 3. Results

All 65 patients included in the initial evaluation, the demographic characteristics of which are reported in Table 1, were reevaluated at 12 months.

According to the initial assessment, the prevalence of depression and anxiety remains high even after 12 months, with a tendency to increase anxiety and depression severity.

**Figure 1:**
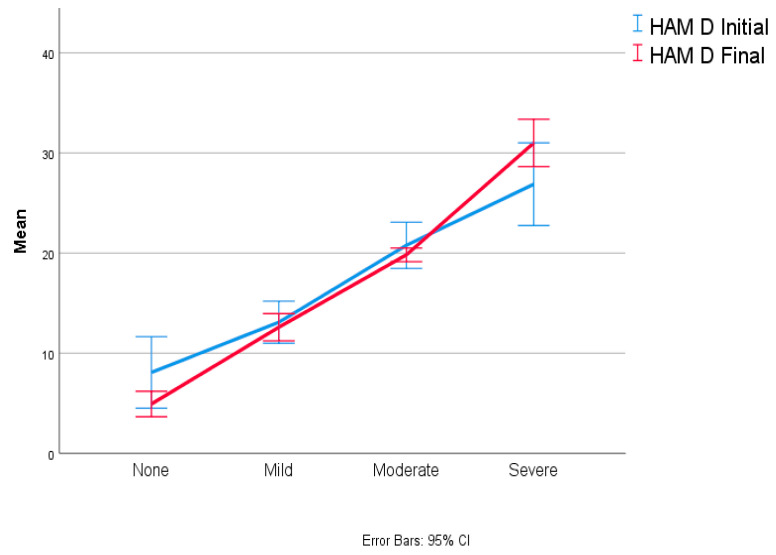
Depression evolution at one-year follow-up.

Out of the 56 patients who reported depression at the initial evaluation, 53 patients (80%) still declared a degree of depression after 12 months. Patients with severe and moderate symptoms, 8 (12.31%) and 22 patients (33.85%), respectively, remained constant compared to the first evaluation. However, the number of patients with mild symptoms decreased from 26 (40%) at initial evaluation to 22 (33.85%) at 12 months. Therefore, only four patients from the first assessment reported that their depressive symptoms had been resolved (Figure 1).

Regarding the reported scores, there was a decrease in the mean and standard deviation from 16.38 (7.98) to 15.77 (8.22), though this result is without significant statistical significance (*p* = 0.311, F (1.64) = 1.044).

**Figure 2:**
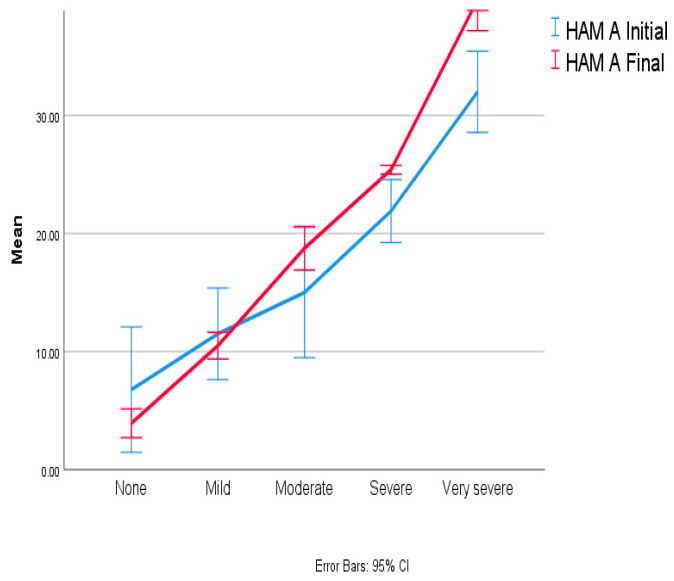
Anxiety evolution at one-year follow-up.

During the initial evaluation, 98.46% of patients presented symptoms of anxiety, whereas depression was less common. After a 12-month assessment, the prevalence of anxiety remained the same, but the severity of anxiety symptoms had increased. Therefore, the number of patients with very severe and severe anxiety symptoms at the 12-month evaluation rose from 16 (24.62%) to 22 (33.85%) and from 6 (9.23%) to 9 (13.85%), respectively. Due to the increased severity of anxiety in the reported scores, there was a decrease in the number of patients reporting mild anxiety from 32 (49.23%) initially to 21 (32.31%) after one year. (Figure 2).

The mean value of the anxiety increased by 2.91 (2.12) points, as depicted in Figure 2. The ANOVA results identified a significant statistical difference, with *p* = 0.006 with F (1,64) = 8.049 and CI 0.086 to 4.96.

Our statistical analysis of the data (linear regression) highlighted a crucial finding, indicating that the progression of anxiety at the 12-month mark is significantly impacted by the initial level of depression (R^2^ = 0.536). Also, considering the value of the unstandardized coefficient (B), the results (shown in Table 2) suggest that the anxiety level increased by 1.234 units for each point scored on the depression assessment scale at the beginning of the study, while considering the evolution, the value rises to 1.303 units/point of initial depression evaluation. This finding underscores, once more, the critical importance of recognizing and treating mood disorders in individuals with SLE.

Regarding the possible mechanisms involved in the manifestation of depression and anxiety in our SLE patients, an initial correlation test revealed a significant correlation (*p* < 0.01) between depression and the presence of anticardiolipin antibodies (0.322), LA (0.404), and anti-ribosome P antibodies (0.427). Strong correlations (*p* < 0.05) with ICAM-1 (0.286), low C4 (0.256), and anti-SM antibodies (0.306) were identified. Regarding anxiety, the initial data showed a robust correlation (*p* < 0.01) with positivity for LA (0.330) and anti-ribosome P antibodies (0.312, *p* < 0.05), as well as low C3 (0.263, *p* < 0.05) and low C4 (0.293, *p* < 0.05), and anti-SM antibodies (0.247, *p* < 0.05).

During the initial evaluation of our study group, nonparametric tests were conducted for HAM A and HAM D. The results showed that SLE patients with positive markers for anti-ribosome P, lupus anticoagulant (LA), anticardiolipin antibodies (ACL), and anti-beta2 glycoprotein (ANTI B2) experienced more pronounced depression and anxiety compared to those with negative biomarkers. However, insignificant differences were observed in anxiety, depression, disability, and quality of life between participants with positive and negative statuses for other analysed biomarkers such as anti-SM, DNA DC, ICAM positive, ANA, and Anti-RO.

In terms of evaluating the possible factors impacting the 12-month evolution of depression and anxiety, in our SLE patients’ predictors are the presence of LA and anti-ribosome P antibodies, suggesting that they influence the evolution of depression and anxiety during NPSLE manifestation and, again, the fact that a complex vascular-inflammatory dual mechanism is involved. The results regarding specific biomarker predictors are represented in Table 3.

Regarding disease evolution assessed through BILAG, the linear regression results suggested that lupus disease activity increases with disability (WHODAS) and anxiety (HAM A), with R2 = 0.503 (*p* < 0.001) and R2 = 0.504 (*p* = 0.023), respectively. Regarding SLE evolution, assessed by moderate disease activity and reflected by the SLEDAI INDEX FLAIR, the linear regression results suggested that it was influenced at one-year follow-up by the WHODAS score (R2 = = 0.229, *p* ≤ 0.0001, B (CI) 0.018 (0.01–0.02)), while the Severe Activity at SFI was influenced by the initial corticosteroid background therapy dose (R2 = 0.115, *p* = 0.006, B (CI) 0.025 (0.01–0.04)). Furthermore, our linear regression analysis (Table 4) on specific SLE disease activity (except for SLICC ACR) and measured biomarkers suggested a heterogeneity of factors influencing SLE manifestation over one year.

The significant results of the ANCOVA test on anxiety and depression follow-up regarding SLE-specific medication, as well as neuropsychiatric treatment, are shown in Table 5. Antidepressive therapy did not statistically significantly influence either anxiety or depression evolution.

In the study, 30 participants did not receive steroid therapy for cortisol treatment, while 35 participants received an average dose of 7.5 mg daily. However, after conducting an ANCOVA analysis, it was found that there was no significant difference between the doses of cortisol and the evolution of depression (*p* = 0.929) or anxiety (*p* = 0.858). Only six cases of severe SLE manifestation (defined by the SFI) were encountered during the study. These cases were treated by increasing the cortisone dose to a maximum of 30 mg/day and rapidly tapering by decreasing the dose at 7 seven days. Therefore, exposure to doses above 20 was not longer than two weeks. The evaluation of depression and anxiety was carried out through the use of questionnaires for all aspects except for the period of exacerbation and cortisol dose increase.

For a secondary analysis, another ANCOVA was performed on subjects who received steroid therapy and those who did not. The results showed that there were no significant differences between the two groups at one-year follow-up, considering the cortisol medication as influencing anxiety (*p* = 0.746) or depression (*p* = 0.257). Of all the study participants, 35 received steroid therapy, accounting for 53.85% of the sample.

Therefore, regarding the results of different types of medication and depression and anxiety manifestation at one year of follow-up, the results suggest that different combinations of SLE medication, like Methotrexate and Plaquenil, can decrease the level of anxiety and depression significantly compared with other pharmaceutical therapies, and they also suggest that anxiolytic treatment can influence the level of depression. The results of our ANCOVAs and linear regression analysis do not suggest that neuropsychiatric therapy has any influence or predictive effect on SLE anxiety symptoms.

## 4. Discussion

As demonstrated in our prior research [15] and substantiated by the existing literature, there is a high prevalence of depression and anxiety in individuals with lupus, exerting a significant impact on affected patients. A noteworthy finding deriving from the current study is the revelation that the prevalence of depression and anxiety persists at elevated levels even after a one-year monitoring period, despite adherence to psychiatric protocols and background therapy. In the general population, depression also persists over time, as shown in previous studies [27,28].

Moreover, at one year of evolution, a notable elevation in the severity of anxiety was observed, alongside the persistently high prevalence of both anxiety and depression, albeit with limited clarification. These findings can be attributed to the fact that the one-year evaluation occurred amidst the COVID-19 pandemic. Data in the literature further support the significant impact of the pandemic on mood disorders, providing context for these observations [29,30].

In a study conducted by Make and colleagues in 2011, it was observed that the phenomenon of anxiety manifestation in patients with SLE could be anticipated based on factors such as depression manifestation, a high cumulative dose of glucocorticoids, and the use of other routine medications. Furthermore, the intensity of depression emerged as a predictor for both the gravity and existence of anxiety. Consequently, individuals with lupus manifesting symptoms of anxiety should undergo concurrent and thorough assessment for potential coexisting depression [31].

Some studies emphasize how depression may function as an independent risk factor in the realm of general healthcare [32].

Conversely, another study aimed to develop a risk nomogram for the prediction of depression likelihood in SLE patients; this investigation unveiled that marital status, education, social support, coping strategies, and anxiety serve as predictive factors for depression in individuals with SLE [33].

Very recent research has revealed that genetically predicted SLE might be associated with a reduced risk of depression [34]. This discovery has prompted a new perspective, suggesting that heightened depression in SLE patients might be attributable to factors that can be modified rather than inherited.

Notable SLE-specific factors, as reported in the literature, encompass pain and fatigue, heightened disease activity, and the involvement of the musculoskeletal and skin systems [35,36].

Many factors can influence modifiable elements, such as self-esteem and coping mechanisms. However, data regarding the evolution of these mood disorders and possible associated factors are very scarce.

Several researchers have focused on exploring the primary causative factors and genetics, and their investigations have concluded that specific genes, such as the *FKBP5* gene, might be determinants of psychological disorders in individuals with SLE [37]. However, despite these indications, there has been no conclusive confirmation of this.

On the other hand, a genetic association has also been observed in other chronic diseases, and one specific study indicated the implication of the *FKBP5* gene in response to antidepressants [38,39]. While studies on anxiety in this context are limited, some reports suggest an association between anxiety and the presence of the *FKBP5* gene [40,41].

Perhaps the involvement of this gene in the pathogenesis of depression and anxiety explains the lack of response to antidepressant treatment in our group of patients, but the improvement of anxiety after antidepressant treatment has been shown.

In previous research, it was reported that depression and anxiety tend to increase with higher SLE disease manifestation. Patients with SLEDAI scores exceeding 8.5 are more prone to experiencing mental disorders, necessitating supplementary awareness of their mental health [42], while a comprehensive international cohort study determined that, over time, there was no discernible link between SLE disease activity and the occurrence of mood disorders [43]. The results of our linear regression analysis show that lupus disease activity reflected on BILAG is linked with the severity of anxiety and disability assessed with WHODAS 2.0.

An essential factor to examine is the prevalent use of cross-sectional designs in many recent studies, which constrains the interpretation of findings since hypotheses extending beyond a fundamental connection between SLE and mental health are difficult to assess. Through conducting longitudinal studies on SLE evolution with flares and periods of controlled disease, our understanding of the association between disposition conditions and SLE could be enhanced. A longitudinal study design enables an examination of potential variations in the interactions between mental health and SLE among different patients. There are few longitudinal data in the literature; one study published in 2002 shows that at 40 weeks, evolution scores for depression and anxiety align with shifts in patients’ evaluations of the activity of their SLE, and there are no data to support the hypothesis suggesting that psychological distress leads to heightened SLE activity [44]. Similar data were obtained in a study conducted over the course of six months in 2009 [45].

The most extended observation period for mood disorders in SLE was 48 months, and this study’s results emphasize that the level of SLE disease activity was associated with depression scores in the multivariate analysis. Still, no significant association was evident in the univariate analysis [19].

In our study, neuropsychiatric treatment had no influence on anxiety and depression evolution; one frequently cited cause could be non-adherence to medical recommendations, which has been observed in primary care settings, where 41% of individuals prescribed antidepressants do not take them [46], similar to data in SLE studies where non-adherence to prescribed treatment regimens has been estimated in up to 46% of patients [47].

An alternative explanation for the constrained efficacy of prescribed SSRI/SNRIs might be their influence on sleep quality; these drugs have been linked to deterioration in sleep quality, a common problem affecting at least half of individuals with SLE [48].

Interestingly, despite the impact that anxiolytics have on patients’ sleep, anxiolytic treatment demonstrates a favorable effect on depression. Similar data show that patients with depressive symptoms could benefit from receiving anxiolytic treatment and that this treatment option should be considered as a way to augment SSRI therapy, but only for a short time, due to the high risk of dependence and cognitive impairment [49,50,51,52].

Regarding response to treatment, the results of a recent longitudinal study indicated that among patients with SLE, depression persists even after interventions targeting pain, mood disorders, and SLE disease [19]. The findings on the rise in depression and anxiety indices highlight the need for further investigation. Despite being one of the few studies to track the progression of these disorders over a year, offering insights into their impact, it appears that these conditions persist and that patients only partially respond to psychiatric treatments. They may progress somewhat independently of Systemic Lupus Erythematosus (SLE), with their underlying causes being not fully comprehended. This suggests the possibility that certain conditions in SLE patients may develop independently, similar to those in the broader population. As part of the background treatment for Systemic Lupus Erythematosus (SLE), our data show that combining Methotrexate and Plaquenil may offer additional benefits by potentially addressing inflammation and thrombotic risk.

This study has several limitations. First, there were few cases, a limited follow-up duration, and a lack of a control group. Subjects with major psychiatric manifestations were not assessed due to the exclusion criteria. Therefore, the results might not be representative of all NPSLE patients. Furthermore, cognitive impairment levels were not investigated.

## 5. Conclusions

After 12 months of careful monitoring and targeted treatment tailored for both SLE and NPSLE in our SLE patients, the prevalence and severity of depression and anxiety remained notably elevated. The presence of specific biomarkers such as LA and Anti-Ribosomal P antibodies appears to be predictive of the persistence of both depression and anxiety, implying that a complex dual inflammatory–thrombotic mechanism is the underlying pathogenic determinant.

Furthermore, this investigation’s findings suggest that while anxiety levels tend to escalate over time, they do not exhibit a direct correlation with SLE activity. The results indicate that the levels of anti-nuclear antibodies and PAI-1 biomarkers can predict SLE disease activity at one-year follow-up. Specific SLE medications like Methotrexate and Plaquenil appear to lower anxiety and depression scores. Furthermore, anxiolytic therapy seems to reduce depression but have no impact on anxiety disorder, which remains an essential issue in NPSLE treatment. In conclusion, this study emphasizes the necessity for further research and the development of more effective therapeutic interventions for NPSLE. Understanding the intricate interplay between SLE and neuropsychiatric symptoms is crucial for improving patient outcomes. There is a clear need for more robust longitudinal studies to explore the multifaceted nature of SLE and its impact on mental health, aiming to enhance the quality of life for those affected by this complex autoimmune disease.

## Figures and Tables

**Table 1:** Patients’ characteristics at inclusion (n = 65).

Characteristic	Mean ± SD/Percent
Age	51.48 ± 13.85 [15]
Active smoking	15 (23.07%)
Non-smoking	50 (76.92%)
BMI	26.6963 ± 6.39
HBP	50 (76.92%)
Dyslipidemia	52 (80%)
Diabetes mellitus	19 (29.23%)
SLE years	12.55 ± 8.10
SLE treatment	
Associated corticotherapy	33/50.76%
Only corticotherapy	3/4.62%
HCQ	54/84.61%
Methotrexate	1/1.54%
Azathioprine	1/1.54%
Micofenolatmofetil + HCQ	2/3.08%
Azathioprine + HCQ	3/4.62%
Methotrexate + HCQ	1/1.54%
SLE major clinical features/organ involvement	
Mucocutaneous	53/80%
Musculoskeletal	65/100%
Serositis	46/70.77%
Kidney	18/27.69%
Cardiac	17/26.15%
Neurologic and neuropsychiatric involvement	35/53.85%
Hematological	59/90.77

BMI = Body Mass Index, HBP = High Blood Pressure, SLE = Systemic Lupus Erythematosus, HCQ = Plaquenil/hydroxychloroquine.

**Table 2:** Anxiety and depression follow-up influences.

Variable	Association/R^2^	B (CI)	*p*
HAM A Final	HAM D Initial/0.536	1.303 (1–1.61)	<0.0001
HAM D Final	HAM A Initial/0.339	0.402 (0.26–0.54)	<0.0001
HAM D Initial	HAM A Initial/0.664	0.538/(0.44–0.63)	<0.0001
HAM A Initial	Ham D Initial/0.664	1.234 (1.01–1.46)	<0.0001

HAM A = Hamilton Anxiety Scale, HAM D = Hamilton Depression Rating Scale, B = unstandardized coefficients, CI = Confidence Interval.

**Table 3:** Specific biomarker predictors for NPSLE manifestation at one-year follow-up.

Assessment Scale	Biomarkers	R^2^	B	*p*
HAM A Initial	LA	0.108	6.78 (1.88–11.67)	0.007 [15]
	C4	0.165	−0.37 (−0.72–0.01)	0.044 [15]
HAM A Final	LA	0.149	9.40 (3.71–14.97)	0.002
	Anti RIB P/	0.096	0.091 (0.02–0.16)	0.012
Differences of HAM A (Final-Initial)	C4	0.156	0.41 (0.17–0.65)	0.001
	D-dimers	0.234	0.01 (0–0.02)	0.015
HAM D Initial	Anti RIB P/	0.183	0.070 (0.33–010)	<0.001 [15]
	PAI	0.258	2.949 (0.60–5.30)	0.014 [15]
Ham D Final	Anti Rib P	0.336	0.098 (0.06–0.13)	<0.001
	LA	0.396	3.755 (0.74–6.77)	0.015
Differences of HAM D (Final-Initial)	D-dimers	0.104	0.008 (0.0–0.001)	0.009

HAM A = Hamilton Anxiety Scale, HAM D = Hamilton Depression Rating Scale, LA = lupus anticoagulant, C4 = complement 4, Anti Rib P = anti-ribosomal P autoantibodies, PAI = plasminogen activator inhibitor.

**Table 4:** Disease activity evolution and influences at one-year follow-up.

Assessment	Biomarkers	R^2^	B	*p*
SLEDAI	Anti Rib P	0.294	0.030 (0.02–0.04)	0.000
	ANA	0.405	0.03 (0–0.01)	0.001
	LA	0.466	1.247 (0.30–2.20)	0.011
	P-selectin	0.518	−0.029 (−0.05−0.01)	0.013
BILAG A	ANA	0.098	0.01 (0)	0.011
BILAG B	PAI 1	0.192	0.497 (0.25–0.74)	0.000
	C3	0.252	−0.11 (−0.02–0)	0.017
	ICAM 1	0.299	0.003 (0–0.01)	0.027
	CRP hs	0.337	0.815 (0.05–1.58)	0.037
Moderate Activity	PAI	0.077	0.216 (0.04–0.39)	0.016
	C3	0.127	−0.007 (−0.1–00)	0.037
Severe Activity	ANTI SM	0.158	0.030 (0.01–0.005)	0.001
	ANA	0.236	0.01 (0)	0.014

SLEDAI = Systemic Lupus Erythematosus Disease Activity Index, BILAG = British Isles Lupus Assessment Group, Anti Rib P = anti-ribosomal P autoantibodies, ANA = anti-nuclear antibodies, LA = lupus anticoagulant, C3 = complement C3, ICAM-1 = intercellular adhesion molecule, PAI = plasminogen activator inhibitor, CRP hs = high-sensitivity C-reactive protein, ANTI SM = anti-Smith antibody.

**Table 5:** ANCOVA results regarding the influences of different medication types on anxiety and depression in SLE patients.

Groups and Therapy	Group Therapy (N)	Mean (CI) of Estimates	R^2^	*p*
Depression and NPSLE medication	Anxiolytic (24)	13.65 (11.46–15.85)	0.698	0.024
	No therapy (41)	17.01 (15.42–18.59)		
	Anxiolytic and antidepressant (20)	14.13 (11.51–16.74)	0.734	0.003
	Antidepressant (7)	21.18 (17.48–24–87)		
	Azathioprine+ HCQ (3)	20.89(15.80–25.98)	0.745	0.024
	Methotrexate+ HCQ (1)	3.87(−4.96–12.69)		
Depression and SLE Medication	Azathioprine+ HCQ (3)	20.89 (15.80–25.98)	0.075	0.030
	Methotrexate+ HCQ (1)	3.87 (4.96–12.69)		
Anxiety and SLE Medication	None (3)	30.44 (22.74–38.15)	0.804	<0.001
	Methotrexate+ HCQ (1)	−9.77 (–23.29–3.75)		
	Azathioprine (1)	23.20 (9.76–36.64)		0.019
	Methotrexate + HCQ (1)	−9.77 (–23.29–3.75)		
	Methotrexate (1)	45.10 (31.75 –58.45)		0.017
	Mycophenolate + HCQ (2)	16.00 (6.37–25.62)		
	Methotrexate (1)	45.10 (31.75 −58.45)		0.023
	HCQ (54)	21.92 (20.11–23.74)		
	Plaquenil (54)	45.10 (31.75 –58.45)		<0.001
	Methotrexate+ HCQ (1)	16.00 (6.37–25.62)		
	Azathioprine + HCQ (3)	21.75 (14.03 –29.47)		0.03
	Methotrexate + HCQ (1)	−9.77 (–23.29–3.75)		

NPSLE = Neuropsychiatric Systemic Lupus Erythematosus, SLE = Systemic Lupus Erythematosus, HCQ = Plaquenil/hydroxychloroquine.

## Data Availability

Data are available upon request.
